# A case of celiacomesenteric trunk in a Tanzanian man

**DOI:** 10.1186/1756-0500-6-341

**Published:** 2013-08-29

**Authors:** Mange Manyama, Anthony Lukanima, Ainoli Gesase

**Affiliations:** 1 Department of Anatomy and Cell Biology, Catholic University of Health and allied Sciences, Mwanza, Tanzania; 2 College of Health and Allied Sciences, University of Dodoma, Dodoma, Tanzania

**Keywords:** Celiac artery, Superior mesenteric artery, Variations

## Abstract

**Background:**

Variation in the branching patterns of the three major arteries that supply the digestive system may occur due to different embryological mechanisms.

**Case presentation:**

The present case report describes the celiac artery and the superior mesenteric artery originating from the abdominal aorta through a common trunk. The celiac artery in turn gave rise to the splenic artery and a common trunk for common hepatic and left gastric artery. The superior and inferior mesenteric arteries had normal branching patterns.

**Conclusion:**

Awareness of these variations on the part of the surgical team before surgery can help avoid iatrogenic arterial injury.

## Background

The celiac artery and the superior and inferior mesenteric arteries are the unpaired branches of the abdominal aorta that provide blood supply to most of the abdominal viscera. The celiac artery (CA) supplies structures derived from embryonic foregut namely abdominal esophagus, stomach, superior half of the duodenum, liver and pancreas [1]. The superior mesenteric artery (SMA) supplies adult derivative of the embryonic midgut, including the whole of the small intestine from superior part of duodenum to the midtransverse colon. The inferior mesenteric artery (IMA) supplies adult derivative of the embryonic hindgut namely the left third of the transverse colon, descending colon, sigmoid colon and most of the rectum [1]. Several anatomic and radiological descriptions of variations involving these arteries have been described in the literature, including the common trunks and anastomoses between CA and SMA, intermesenteric arcade between SMA and IMA or the celiac-bimesenteric trunk which consists of a common arterial trunk between the CA, SMA and IMA [2,3]. These variations and others that involve the gastrointestinal tract vascular system are likely due to unusual embryological development of the ventral splanchnic arteries. Presence of the celiacomesenteric trunk of which its injury can lead to lethal effects to the individual however, is not common [4]. The rare occurrence of celiacomesenteric trunk variation has been stated to be between 1% and 2.7% [5]. Various anatomical variations in the coeliac trunk–hepatic arterial system and the renal arteries have also been reported in patients undergoing multidetector CT (MDCT) angiography of the abdominal aorta [6]. Presence of vascular variations in any single organ therefore could indicate the possibility of variations in the vascular structure of other organs. Elsewhere, the classical configuration of the celiac trunk has only been detected in about 72% of individuals while hepato-splenic trunk, hepato-gastro-splenic trunk and gastro-splenic trunk was detected in 50.4%, 19.4% and 2.3% of individuals respectively [7]. Use of Multidetector-row CT (MDCT) which provides high-quality 3D-reconstructed images and allows non-invasive assessment of normal anatomy and anatomic variants of celiac trunk has therefore been recommended [7]. Variations in the branching pattern of the major gastrointestinal vascular system have also been reported including a four-branched coeliac artery that includes the left gastric, common hepatic, splenic and pancreatic-duodenal arteries (instead of three) [8].

These variations are important clinically since awareness of such variations on the part of the surgical team before surgery can help avoid iatrogenic arterial injury, particularly in centers where angiographic studies are not performed regularly.

## Case presentation

**Figure 1:**
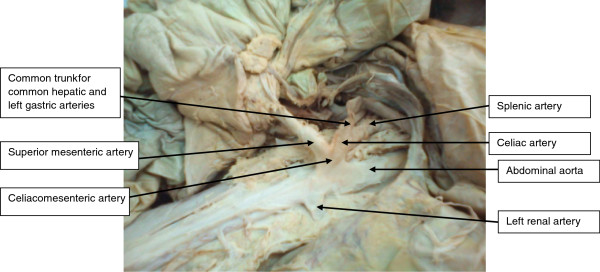
Photograph shows the celiacomesenteric trunk with its branches.

The present case report describes variation in the origin of the celiac artery and superior mesenteric artery in a 36-year old Tanzanian male cadaver during dissection sessions for medical students in the dissection laboratory of the Catholic University of Health and allied Sciences. Medical records prior to his death indicate that his weight was 69 kg and his height was 1.65 m. Further records shows that he had no past medical history of chronic gastrointestinal or vascular diseases and the cause of death was not due to illness involving either of the two body systems. The cadaver was formalin-fixed and dissection of the abdomen was performed first by opening the abdomen through several incisions on the anterior abdominal wall. In order to expose the CA, SMA and IMA, the transverse colon and greater omentum were mobilized superiorly over the coastal margin, while the coils of the jejunum and ileum were moved to the left of the abdomen. The CA and the SMA were found to originate from the ventral surface of the abdominal aorta on a common trunk (celiacomesenteric) about 2 cm below the aortic hiatus (Figure 1). Shortly after branching from the short celiacomesenteric trunk, the celiac artery divided into the splenic artery and a common trunk for common hepatic and left gastric artery. The SMA on the other hand gave rise to several branches including the intestinal branches, middle and right colic arteries, and the ileocolic artery. The IMA artery was found to originate from the ventral aspect of the abdominal aorta about 4 cm above the bifurcation of the aorta with normal branching pattern. The variation described above was not detected prior to death partly due to the fact that no radiological investigation was done.

## Discussion

During embryological period, three sets of arteries for the trunk originate from the aorta. The anterior arteries supplies the digestive system, posterior arteries are parietal and lateral arteries supplies the urogenital system. In human embryos, the paired vitelline (primitive intestinal) arteries are redirected to supply various regions of the gut tube as the embryo folds cephalocaudally and laterally to form the gut tube [9]. The CA form from the more cranially placed vitelline vessels and serves the foregut, middle placed vitelline vessels forms the SMA which supply the midgut; and distal vessels form the IMA supplying the hindgut [9]. In majority of individuals, the CA originates from the abdominal aorta just below the aortic hiatus, the SMA originates from the aorta about 1 cm below the celiac trunk at the level of L1-L2 vertebral disc, and the IMA arises 3–4 cm above the aortic bifurcation [1].

The present case describes a finding of the celiac artery and superior mesenteric artery originating from the anterior surface of the aorta through a common trunk (celiacomesenteric trunk). This type of vascular variation have been reported previously [2,10]. The CA, SMA and IMA develop from the 10th, 13th and 21st or 22nd metameric intestinal (vitelline) arteries that supply the yolk sac of the embryo [3]. Most of the other metameric arteries and the Tandler’s anterior longitudinal anastomosis which connects them regress during the evolution of the embryo [11]. In some cases, the persistence of the anterior anastomosis or regression of one of the primitive arteries that form the CA, SMA and IMA may explain some of the variations observed in the adult [12]. Embryologically therefore, the occurrence of celiacomesenteric trunk can be explained by regression of the 10th root and persistence of both the 13th root and the anterior anastomosis. The diagnosis celiacomesenteric trunk has often been reported during autopsy or accidentally during angiography or abdominal computed tomography scanning [5,13]. This anomaly have been reported to be associated with clinical conditions like chronic occlusive disease, compression by abdominal aorta aneurysm [14], celiac compression syndrome [15], or rarely large gastrointestinal infarction due to thrombosis of the celiacomesenteric trunk [4]. Thrombosis of the celiacomesenteric trunk can lead to serious gastrointestinal complications including necrosis of the gastro-intestinal tract (from the stomach up to the first third of the transverse colon), ischemia of the liver and infarction spleen infarction [4]. These complications can easily lead to death.

Several other variations involving these arteries and explanation for their occurrence have been provided. The arcade of Buhler that connects the CA and SMA is explained by persistence of the 10th and 13th primitive arteries associated with the persistence of the ventral anastomosis between these two arteries [10]. Existence of the intermesenteric arcade that connects the SMA and IMA have been reported to be due to the persistence of the anterior anastomosis between the 13th and 21st or 22nd primitive intestinal arteries [3]. Celiac-bimesenteric trunk where by all three arteries supplying the abdominal digestive organs converged into one trunk has also been reported [3]. Existence of this variant have been postulated to be due to the regression of the 10th and 21st or 22nd primitive arteries, with persistence of both the 13th root and the anterior anastomosis, that provides a common trunk of origin for the CA, SMA, and IMA [3].

## Conclusion

In conclusion, we found the celiac artery and superior mesenteric artery originating from the common trunk, which previously have been called the celiacomesenteric trunk. Knowledge on the existence of vascular variations or anomalies regarding specific region of the body helps during planning of surgical intervention and also prevent mistakes due to lack of awareness. This is particularly important in centers where angiography are not done regularly.

## Consent

A written consent was obtained by the cadaver’s next of kin for publication of the article. A copy of the written consent is available for review by the Editor-in-Chief of this journal.

## Competing interests

The authors declare that they have no competing interests.

## Authors’ contributions

MM did the dissection, obtained the photos, wrote the draft of the manuscript and obtained the written consent. AL performed the literature review and participated in the manuscript writing. AG helped to the final writing of the paper. All authors read and approved the final manuscript.

## Authors’ information

MM is a Medical doctor and Senior Lecturer in Anatomy and Cell Biology. AL is a Medical doctor and teaching assistant in the Anatomy. AG is a Professor of Anatomy.
